# Investigations on Five PMMA Closed Types of Piezo Actuators as a Cooling Fan

**DOI:** 10.3390/polym15020377

**Published:** 2023-01-10

**Authors:** Rong-Tsu Wang, Jung-Chang Wang

**Affiliations:** 1 Department and Graduate Institute of Information Management, Yu Da University of Science and Technology, Miaoli County 36143, Taiwan; 2 Department of Marine Engineering (DME), National Taiwan Ocean University (NTOU), Keelung 202301, Taiwan

**Keywords:** PMMA, acrylic, actuator, PA, piezoelectric ceramic, thermal analysis

## Abstract

There are five closed types of piezo actuators (closed type of PA, closed PA) as a cooling fan relative to those different PAJs of the previous work (open type of PAJ, open PAJ) for analysis in the present study. Closed PA was composed of circular piezoelectric ceramics (PCs) and acrylic (PMMA) plates and investigated on five different types at operating conditions. The results show that the noise of the closed PA is quieter than that of the open PAJ by about 10 dB. When the closed PA is deposed at a suitable distance of 10 to 20 mm from the heat source, averting sucking back the high-temperature fluids around that, the thermal convection coefficient is above 120% more than that of the conventional rotary fan. The cooling performances of these five closed PAs were evaluated by thermal analysis technique, and the convection thermal resistance of the best closed PA can be decreased by over 15%. In terms of energy consumption, a monolithic closed PA was less than 10% than that of a rotary fan. Among these five closed PAs, the best one has the essential qualities that the diameter of the piezoelectric sheet is 41 mm, the opening length is 4 mm, and the outer opening length is 10 mm. Moreover, the best operating conditions are a voltage frequency of 300 Hz and a release distance of 15 mm in the present study.

## 1. Introduction

Chang et al. [1] utilized a PMMA plate with two circular piezoelectric ceramics (PCs) as an actuation jet named by the open PAJ for dissipating the heat of electronic devices in the previous study. The results showed that the heat convection effect was greater than that of a traditional rotary fan and the power consumption of a single open PAJ was less than 10% of that of a rotary fan. However, a flapping cantilever crossbeam constitution employing the piezoelectric matter for heat dissipation was first achieved in 1978 by Toda [2,3,4,5], in which integrated polyvinylidene fluoride resin 2 (PVF2) as the fan blade. There has been consequential attentiveness forwarded in the polymer of PVDF (PVF2 or polyvinylidene difluoride) based on the best piezoelectric behavior among these trade polymers. PVDF (homo- and co-polymers) is familiarly blended and polymerized between 5 and 160 °C and between 5 and 350 atm. Moreover, piezoelectric fans are thin elastic beams whose vibratory motion is actuated by means of piezoelectric material bonded to the beam. These fans have found use as a means to enhance convective heat transfer while requiring only small amounts of power, which is to quantify the influence of each operational parameter and its relative impact on thermal performance through the vibration frequency and amplitude as well as the geometry of the vibrating cantilever beam [6]. Yorinaga et al. [7] introduced a fan consisting of a piezoelectric bimorph tipped with an additional flexible blade in 1985. The fan case has two air outlets which are directed oppositely each other. The fan with such outlets can supply an airflow of 4 m^3^/h at an applied voltage of 140 V_p–p_, which is suitable for low-voltage operation, and it can supply airflow of 1 m^3^/hr at an applied voltage of 17 V_p–p_. Liu et al. [8] presented an experimental work concerning the thermal performance of piezoelectric fans. A total of six piezoelectric fans with various blade geometries are made and tested. It is found that the heat transfer augmentation of the piezo fan comes from the entrained airflow during each oscillation cycle and the jet-like air stream at the fan tip, yet these two modes are of the same order of magnitude. Based on the dimensionless analysis of the test results for all six fans, a correlation applicable for *x*/*L* = 0 is proposed. The mean deviation is 4.8%, which can well describe the influence of geometrical parameters.

The disclosure and development of micro-generators during the last decades has expressed a fine harvesting powering solution. It has also created a great background in engineering energy microsystems involving fabrication methods, system concepts, and optimal functionality. A brief representation of the major transduction mechanisms employed, namely the piezoelectric, electrostatic, electromagnetic, and triboelectric harvesting concepts. The mechanical structures used as motion translators include the employment of a proof mass, cantilever beams, the role of resonance, unmorphed structures, and linear/rotational motion translators. In this direction, the evolution of broadband electromechanical oscillators and the combination of environmental harvesting with power transfer operating schemes could unlock a widespread use of micro-generation in microsystems such as micro-sensors and micro-actuators [9]. Dau and Dinh [10] reported the numerical and experimental study of a valveless micro blower actuated by a lead zirconate titanate (PZT) diaphragm. The flow rate of the device of an air generator can be up to 0.7 l/m, and the developed back pressure is 300 Pa. Park et al. [11] conducted to analyze the effects of the freestream and simulated the unsteady flow on the 3-D piezoelectric fan. The counter-rotating vortices from the fan tip are more significantly affected by the freestream than the vortices from the fan side. Finally, two critical values of the Strouhal number were found in this study for determining the performance of a vibrating plate within the freestream. Revathi and Padmanabhan [12] proposed a piezoelectric polymer composite-based micropump that is biocompatible and inexpensive. Lead Zirconate Titanate (PZT) powder is dispersed in polyvinylidene difluoride (PVDF) to form a novel PZT/PVDF composite film material, which was used as the actuator. The piezoelectric polymer-based valveless micropump, thus, fabricated is subjected to performance testing. The micropump is found to deliver fluid in small quantities in a controlled manner, and sufficient back pressure and flow rate can be achieved at low applied voltage and frequency. Gil [13] investigated experimentally the heat transfer enhancement of an air-cooled heat sink using multiple synthetic jet actuators. A correlation of thermal resistance as a function of Reynolds number and dimensionless stroke length based on the experimental results was presented and discussed. The synthetic jet piezoelectric air pump is a potential miniature device for electronic cooling. The convective heat transfer coefficient of the synthetic jet piezoelectric pump is 28.8 W/(m^2^⋅°C), which can prove that the device has a better heat dissipation capability [14].

Yoo et al. [15] fabricated various kinds of piezoelectric fans with lead zirconate titanate (PZT-5) raw material for cooling applications having AC 110 and 220 V, 60 Hz sources. The experiments showed the highest values of displacement and wind velocity, 35.5 mm and 3.1 m/s, respectively, at 220 V and 60 Hz. Zhang et al. [16] investigated that synthetic polymer jets driven by cantilever PZT bimorphs were fabricated and their cooling performance on a heat sink fin tip surface. Geometrical parameters of the synthetic jets, including cavity size, cavity depth, orifice size, orifice length, and diaphragm thickness, were optimized for increased jet velocity and high cooling performance using the Taguchi test method. Based on the test results, a synthetic jet with an optimized structure was fabricated. Measurements showed that the optimized jet could produce a peak air velocity of 50 m/s at 900 Hz from a round orifice 1.0 mm in diameter. The power consumption of the jet in this condition is 0.69 W, and the total mass is 6 g. Using the optimized synthetic jet, a heat transfer coefficient of 576 W/m^2^K was achieved on the fin tip, indicating an increase of 630% over natural convection values. Wang et al. [17] utilize an acrylic (PMMA) plate with circular piezoelectric ceramics (PCs) as an actuator to design and investigate five different types of piezo actuation jets (PAJs) with operating conditions. The present study greatly improves the constructions of the open PAJ, which employs the circular piezoelectric ceramics (PCs) into the acrylic (PMMA) plates preventing the noise and enhancing the performances of the closed PA as a cooling fan. In addition, only one single circular piezoelectric ceramic (PC) has been applied in the closed PA. In other words, the difference between open PAJ and closed PA was the vibration effect resulting from the position and number of the PC. There are five closed PAs manufactured for various parameters, including the sheet spacing, the size, and the opening area, to set up the performance test methods and conduct cooling experiments on the heat dissipation of high-power LEDs to determine the best closed PA as combining several closed PAs in series. Connecting the closed PAs in series strengthened the overall amount of wind and permitted for the addition or subtraction of closed PAs according to the area of the heat source to effectively control the volume of the closed PA and achieve the best dissipation effect.

## 2. Research Methods

**Figure 1:**
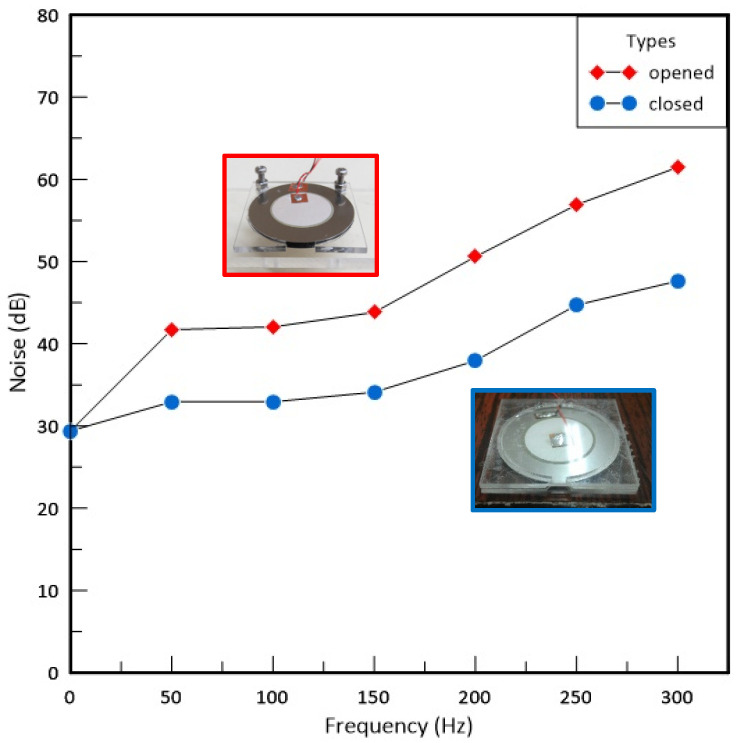
Nosie of PA and PAJ.

**Figure 2:**
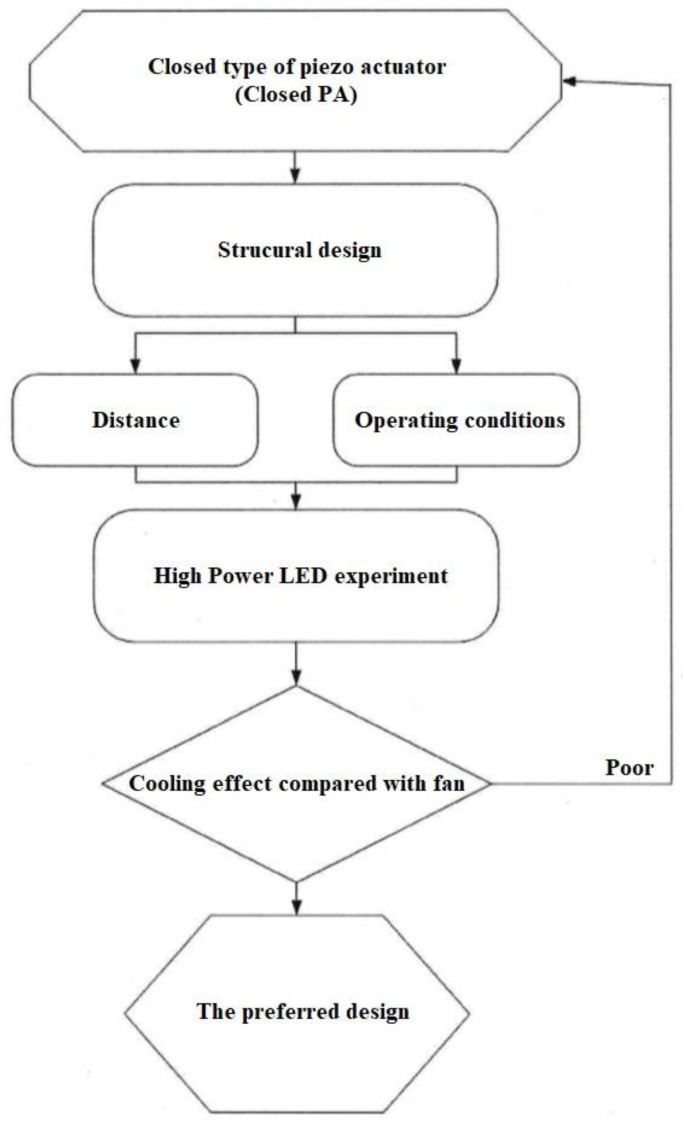
Experimental flow chart.

The closed type of piezo actuator (Closed PA) is composed of a circular piezoelectric sheet and acrylic (PMMA) in the present paper, which the materials of PMMA called ACRYREX^®^ CM-207 with excessive gloss, distinct transparency, and great smoothness were furnished by Chi Mei Corporation (Taiwan, China). The closed-type structural design patterns discussed in this study included the opening size, spacing, and area. Figure 1 exhibits the noise of the closed PA is quieter than that of the open PAJ by about 10 dB. The best performance of the closed PA device was selected through the high-power LED (HI-LED) [18,19,20,21,22,23] experimental process, which can be combined in series to increase the overall air volume and wind speed, and the number of series can be increased or decreased according to the area of the heat source effectively control the volume of the closed PA device and achieve the best heat dissipation performance. The experimental flow chart is revealed in Figure 2, which involves structural design verified by measuring procedures and properties. These performance measurements contained noise, displacement, and wind speed. A closed PA device was investigated in HI-LED thermal performance module experiments through these parameters and through thermal resistance analysis.

### 2.1. Structural Design Patterns

**Figure 3:**
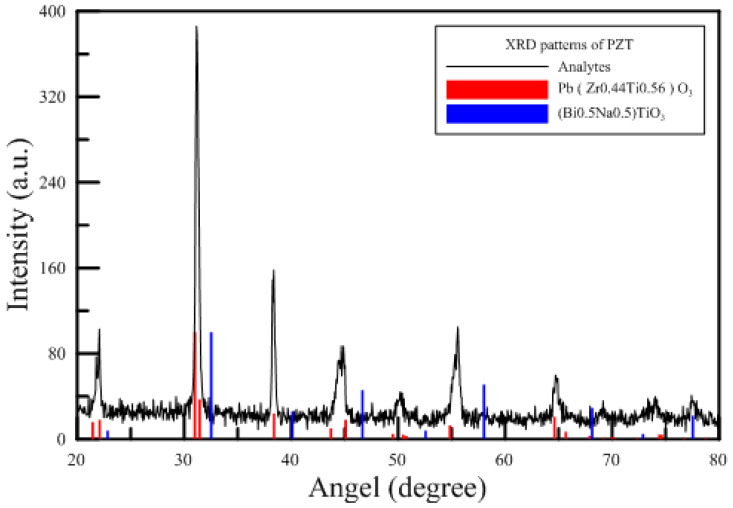
XRD patterns of the present PZT [1].

The Ref. [1] of open PAJ verified the diameter of the piezoelectric sheet of 41 mm, which straightly impacted the performance of the device of the closed type of piezo actuator (Closed PA) as a cooling fan. And the present experiment employed the five PMMA structural design patterns to explore the performance of the closed PA device. The base material of a piezoelectric sheet in this experiment was an iron–nickel alloy; the basic properties were Young’s modulus of 141 GPa and Poisson’s ratio of 0.29. Figure 3 displayed the XRD (X-Ray Diffraction) patterns of the circular piezoelectric sheet of Lead Zirconate Titanate (PZT), which revealed the chemical formula of Pb (Zr_0.44_Ti_0.56_)O_3_ as same as the Ref. [1]. The present study utilized the matter of the PMMA with a density of 1.16 g/cm^3^, a melting point of 135 °C, a glass temperature of 102 °C, thermal conductivity of 0.23 W/(mk), and a tensile strength of 78 MPa, which were fabricated by the skill of the insert injection molding process of the vapor chamber (VCRHCS) [24,25,26,27] as same as the Ref. [1] and the strength can be improved outstandingly and lower the defect of the welding lines.

**Figure 4:**
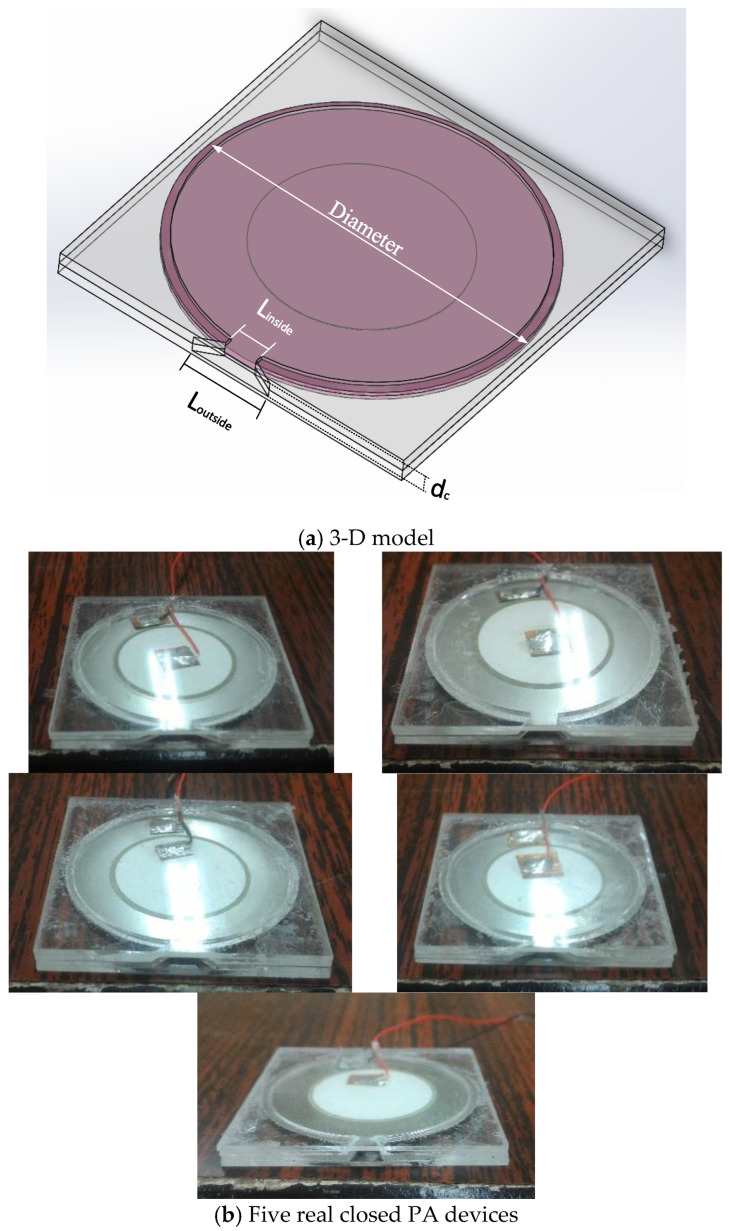
Schematic diagram of closed PA.

Table 1 displays the detailed specifications of a closed PA device, including six parameters. Ref. [1] examined the flared jet channel with an opening length of 4 mm and a spacing of 2 mm. Therefore, the opening part of the closed PA was designed with a gradually expanding flow channel. Fix the inside length (L_inside_) of 4 mm and alter the outside length (L_outside_) from 6 mm, 8 mm, 10 mm, 12 mm, to 15 mm, as shown in Figure 4a. That guided the air volume flow from the cavity to the outside, and the heat dissipation area was increased. All acrylic sheets had 1 mm thickness, which was mainly made in multiple layers for the convenience of subsequent series connection and low cost. The area of the closed PA device was only less than 4% of the current commercial rotary fan. The five tested real closed PA devices are shown in Figure 4b.

#### 2.1.1. Noise

**Figure 5:**
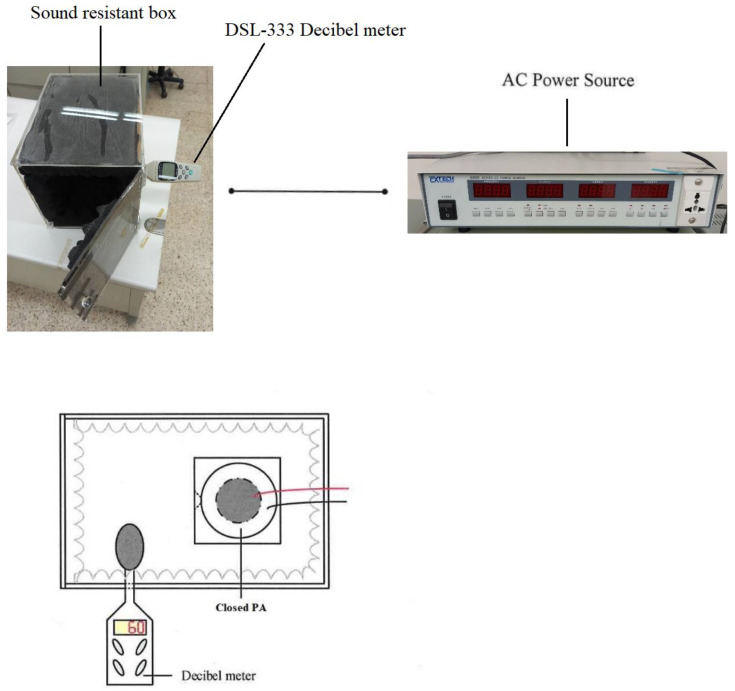
Noise experiment.

Figure 5 exhibited the schematic diagram of the noise experiment of the device of the closed type of piezo actuator (Closed PA) as a cooling fan, in which a sound-resistant box made by hand was adopted in this experimentation. A rotary fan of 12 cm evaluated in the consecutive tests of the sound-resistant box was compared with the closed PA device. The sound-resistant box sheet constructed by acrylic has a thickness of 5 mm and a size of 250 × 250 × 350 mm^3^, and the surface is covered with wave-shaped soundproof cotton at a thickness of 50 mm. The noise was measured by a DSL-333 decibel meter with a measuring scope between 30 and 130 dB according to the 0.1 dB resolution, where the error is within ±1.5 dB, and the frequency response is between 30 Hz and 8 kHz. The rotary fan makes a noise value of 62.4 dB outside the sound-resistant box when yet put inside the box with a noise value of 50.7 dB. Equation (1) revealed the volume of sound correction of the background. The background volume of the experimental room was estimated to be 41.9 dB; however, the value was revised to 50.1 dB according to the standard noise control Equation (1) after correction. This demonstrates that the sound resistance box has well soundproofing ability.

(1) $$L_{0}=10log\left(10^{0.1L_{1}}-10^{0.1L_{2}}\right)$$

*L*_0_: Measured value of the intended sound source;
*L*_1_: Measured value of the total volume of sound;
*L*_2_: Measured value of background volume of sound.

The noise of traditional fans is caused by the wind shearing sound produced by the blade end, the air turbulence, and the bearing rotating friction. The closed PA device uses metal sheets to replace the blades, which can eliminate the need for maintenance and no wind shearing effect. The source of its noise is the sound produced by the vibration of the metal sheet, and the sound intensity depends on the input voltage and frequency. The steps of this experiment are as follows. (1) Confirm that there is no external noise interference and movement of people to control the ambient noise below 45 dB. (2) Insert the decibel meter of DSL-333 into the sound-resistant box and stick soundproofing cotton at the gap to achieve complete sound insulation, and Install the draft shield. (3) Connect the closed PA device to the AC power supply, and the driving voltage and the initial frequency are 30 V and 50 Hz, respectively. (4) The frequency is every 50 Hz as an interval, and the measurement frequency range is between 50 and 450 Hz. (5) Record the measured decibel reading and draw a chart after the experiment.

#### 2.1.2. Displacement

**Figure 6:**
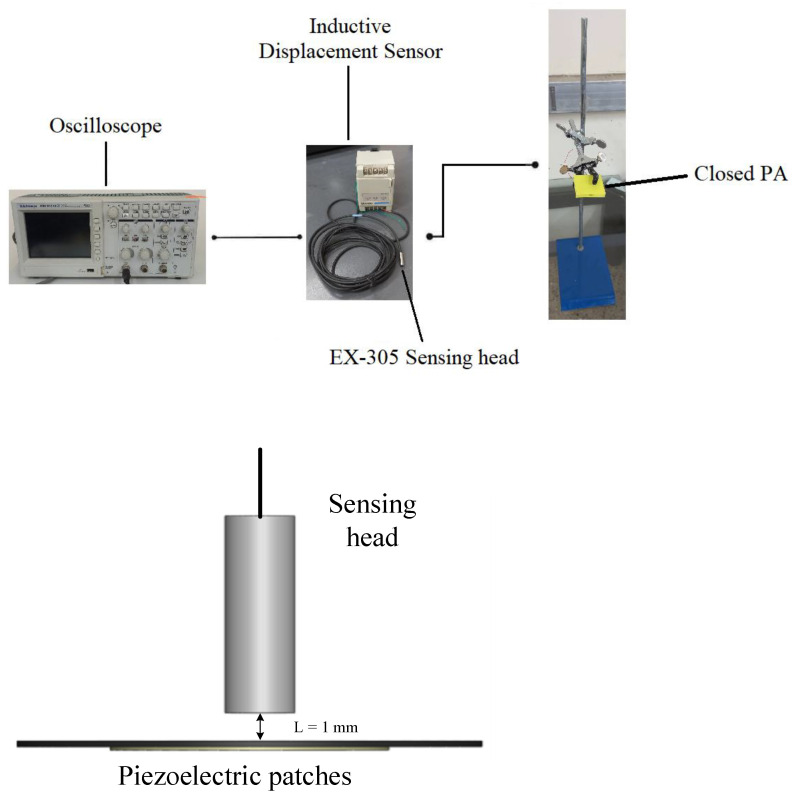
Schematic diagram of the jet path.

In this experiment, the piezoelectric ceramic (PC) sheet is the actuation source, which transfers electrical energy to the metal sheet and converts it into mechanical energy resulting in vibrating the metal sheet and regularly squeezing the air inside the PMMA cavity to form a jet. The displacement of the PC sheet mainly depends on the voltage and frequency by way of employing a three-leg fixture to clip the closed PA device and sensor head, as revealed in Figure 6. The frequency response and resolution for the EX-305 sensing head are, respectively, 18 kHz and 0.4 μm with operating temperatures between –10 °C and 60 °C. L = 1 mm means the measurement distance between the EX-305 and piezoelectric patches. The degree of interference of the sensor head impedance by the metal sheet and the high-frequency magnetic field caused as high-frequency current were converted into the voltage output. Consequently, the voltage signal was transported to a digital oscilloscope to create a waveform, and the displacement or strain of the metal sheet can be evaluated from the amplitude of the Y-axis of the waveform.

The steps of this experiment are as follows. (1) Open a circular hole with a diameter of 5.5 mm in the acrylic below the closed PA device. (2) Connect the closed PA device to the AC power supply with a driving voltage of 30 V and an initial frequency of 50 Hz. (3) Place the sensor head in the circular hole and adjust the measurement distance between them to be 1 mm. (4) Employ a DC power supply to drive the eddy current displacement sensor and connect the output end to a digital wave device. (5) The frequency is every 50 Hz as an interval, and the measurement frequency range is 50~300 Hz. (6) Capture the graph displayed by the digital oscilloscope and convert the voltage value measured by its voltage axis into the displacement. (7) Replace the piezoelectric sheets of different sizes and repeat the above steps.

#### 2.1.3. Wind Speed

**Figure 7:**
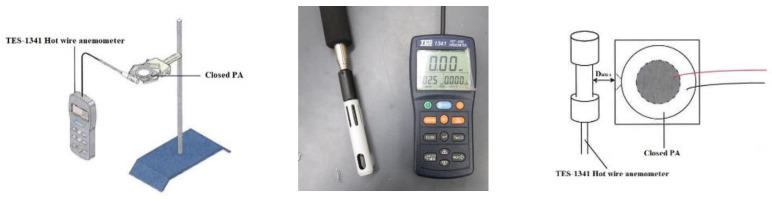
Hot wire anemometer.

The objective of this experiment is to explore the position of the closed PA device from the heat source and find out the best placement between the closed PA and the heat source through this experiment to improve the heat dissipation effect. Generally, the air volume flow rate transmitted by the traditional rotary fan to the heat source is inversely proportional to the placement position, and the wind speed decreases with the increase in the distance. Accordingly, when the distance between them is too close, the flow resistance increases, and the heat dissipation effect may be worse than that of free convection. Thus, it is known that the placement distance is an important parameter that cannot be ignored. In the present experiment, a closed PA device was fixed by a tripod fixture, and the distance D_HWA_ from the hot wire anemometer named TES-1341 was changed at distances of 5, 10, 15, 20, and 25 mm, respectively, to measure the wind speed. The schematic diagram of the wind speed experiment is shown in Figure 7. The measured wind velocity ranges of TES-1341 are from 0 to 30 m/s with a 0.01 m/s resolution and an error within ±3%.

The steps of this experiment are as follows. (1) Calibrate the hot wire anemometer based on the instructions in the manual. (2) Place the hot wire anemometer 5 mm in front of the closed PA device. (3) Connect the closed PA device to the AC power supply; the driving voltage is 30 V, and the initial frequency is 50 HZ. (4) The placement position is every 5 mm as an interval, and the farthest measurement is 25 mm. (5) Change the input voltage frequency under the same placement position, and the frequency is every 10 Hz as an interval, and the measurement frequency range is 50 to 450 Hz. (6) Record the average wind speed measured and draw a chart after the experiment.

### 2.2. Thermal Resistance Network Analysis

The thermal resistance value is mainly used to assess the heat dissipation qualification of the HI-LED package when designing HI-LED heat dissipation. The dimensions and power of this HI-LED are 36 × 34 × 2.6 mm^3^ and 10 W, respectively. The heat dissipation capability of the closed PA device as a cooling fan can also be used to judge by detecting the level of thermal resistance. That is, the larger the thermal resistance value, the poorer the heat dissipation effect, and, therefore, the overall chip temperature is higher. The smaller the thermal resistance value, the better the closed PA device capability. Equation (2) exhibits the definition of thermal resistance.

(2) $$R_{T}=\frac{T_{j}-T_{a}}{W}$$

*R_T_*: Total thermal resistance, *T_j_*: Junction temperature, *T_a_*: Ambient temperature, *W*: Power Dissipation

**Figure 8:**
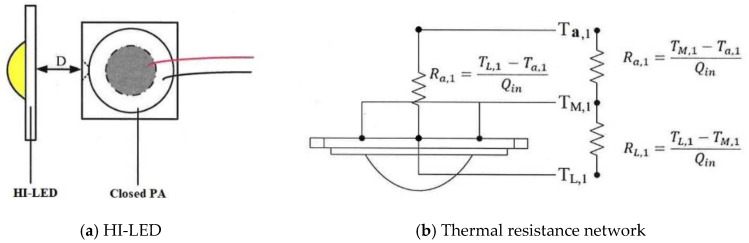
LED Module network.

The thermal performance test methods instructed the cooling experiments in confidence that the closed PA device as a cooling fan could be employed in the heat dissipation of HI-LEDs. Figure 8 displayed this experiment that was two parts. The first part discussed the thermal resistance between natural convection and forced convection for HI-LEDs without fins. The second part was the influence of heat dissipation fins on the same HI-LEDs. The thermal resistance of the present HI-LED can be resolved in two parts based on thermal performance modules, including the thermal resistance of the heat diffusion (*R*_*L*,1_) and the thermal resistance of the natural convection (*R*_*a*,1_), as revealed in Equation (3). The heat diffusion rate is spreader due to the thermal conductivity of the material because of the greater surface area of the LED substrate than the LED heat source such that the diffusion resistance is generated.

(3) $$R_{L,1}=\frac{T_{L,1}-T_{M,1}}{Q_{in}}$$

The thermal resistance *R*_*a*,1_ describes that the temperature difference between the average temperature of the substrate interface (*T*_*M*,1_) and the ambient temperature (*T*_*a*,1_) is divided by the power (*Q_in_*) as shown in Equation (4), in which the energy transmission generated through the density difference between the LED substrate temperature and the air is called free convection. A fan can also be mounted to raise the convection effect resulting in a forced convection phenomenon. The thermal resistance of heat convection exhibits the transfer process that heat capacity dispersed into the ambient via convection.

(4) $$R_{a,1}=\frac{T_{M,1}-T_{a,1}}{Q_{in}}$$

To simplify the diagram of the network analysis, $R_{L,1}+R_{a,1}=R_{T,1}$ of the thermal resistance, $R_{T,1}$ is the total thermal resistance, as shown in Equation (5).

(5) $$R_{T,1}=\frac{\left(T_{L,1}-T_{a,1}\right)}{Q_{in}}$$

## 3. Results and Discussion

The present work observed the attributes of the device of the closed type of piezo actuator (Closed PA) as a cooling fan regarding the heat dissipation capacity with a high-power LED (HI-LED) module. The high temperature around the HI-LED heat source will be reflowed, resulting in raising the chamber temperature and reducing the cooling efficacy when the closed PA device is too close to the HI-LED. The closed PA device possesses a constricted region to enlarge the cooling area and promote the jet stream wind speed, which can crucially lower the thermal convection resistance. These operating situations, structural designs, and positions impact thermal performances and cooling functions. Therefore, we dominated to reform the operating performance and draw the best design of a closed PA device as a cooling fan.

### 3.1. Experiment of Performance Measurement

A piezoelectric sheet is a kind of sound-emitting element that transforms electric power to tone, in which its pitch and intensity depend on the voltage and frequency. The voltage was regularized at the maximum rated voltage of 30 V, and the frequency measurement range was 50 to 450 Hz. Simultaneously, the relationship between the voltage, frequency, and displacement of the metal sheet was discussed.

#### 3.1.1. Noise

**Figure 9:**
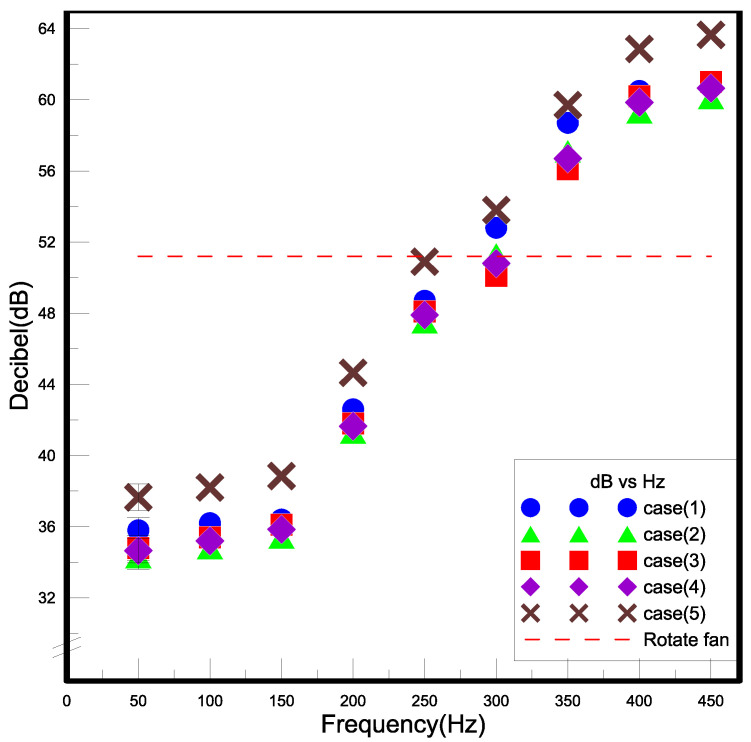
The results of noise measurement.

The present experiment employed a self-made soundproof box, in which the background noise of the lab was 41.9 dB, while inside, the empty soundproof box was 32.8 dB. Figure 9 shows the noise generated by the five cases at 50~450 Hz, in which the growth trends of these five cases were similar. The volume increased sharply after 150 Hz and started to slow down at 350 Hz. The red line segment is a rotating fan. The decibel value measured by the rotating fan in the soundproof box was 51.2 dB, which is similar to the decibel value of the closed PA at 300 Hz. The experimental results showed that the noise generated by the five closed PAs at 300 Hz was similar to that of the rotary fan and was lower than that of the rotary fan when the frequency was 50 Hz to 200 Hz, and the closed PA had no wind shear. If the low-frequency operation was used, the noise could be effectively reduced. After the formula was revised, the noise decibel was shown in Table 2. The results showed that the errors were all within 1.4%, indicating that the self-made soundproof box had a good sound insulation effect and was not disturbed by external background noise. After discussion, the following factors that may affect the noise measurement data and improvement methods were proposed. When the rotary fan rotates, the noise generated by the wind shear effect and bearing friction results in a slight difference from the decibel number marked on the product. The closed PA is fixed on the fixed frame. Due to the acrylic force squeezed to the upper and lower sides, the sound of the welding point on the piezoelectric sheet and the acrylic force colliding with each other can be heard between the fixed jaw and the closed two metal sheets are added to the PA to make the force point even and reduce the noise. There will be some protrusions of the welding wire on the piezoelectric plate, and this protrusion will cause noise when the piezoelectric sheet vibrates and hits the acrylic, so use a thinner wire for welding, and the protrusion will be lower, which can make the probability of impact is reduced, and noise generation is reduced. Coating a layer of resin on the metal sheet can effectively reduce its noise value and can also paste sound-absorbing cotton around it to absorb the sound emitted by the metal sheet.

#### 3.1.2. Displacement

**Figure 10:**
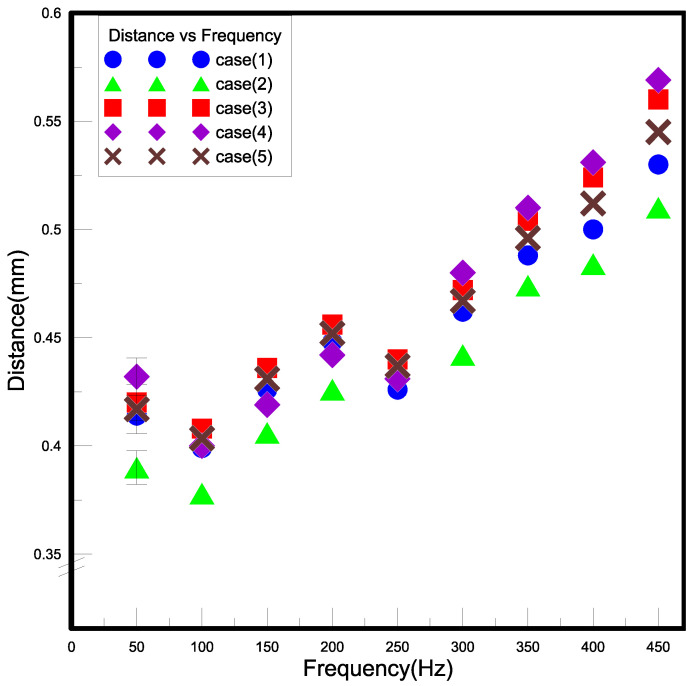
The results of displacement measurement.

The employed adhesives and the attached probes will be moderately not the same in view of hand error as constructing a device of closed PA in this paper. However, the piezoelectric sheets used are all the same, so this part of the experiment mainly finds out the frequency relationship between the piezoelectric sheet deformation and the input voltage. Moreover, it can be deduced from the disclosures that the same types of piezoelectric sheets have similar vibration behaviors. Figure 10 exhibited the relationship between the voltage frequency and the deformation distance of the piezoelectric ceramic metal sheet of the five closed PAs at 50 to 450 Hz. In the data, the displacement of the piezoelectric sheet is slightly different due to the amount of adhesive applied during the production of the closed PA and the human error when fixing the measuring head. The measure of deformation distance increased significantly when the input frequency was above 150 Hz. The amount of displacement and the vibration of the metal sheet is proportional to the input voltage frequency. When the frequency is 100, 250, and 400 Hz, there is a weakening phenomenon in which the natural frequency of the metal sheet does not match the supply frequency, so the metal sheet cannot resonate with the supply frequency.

#### 3.1.3. Wind Speed

**Figure 11:**
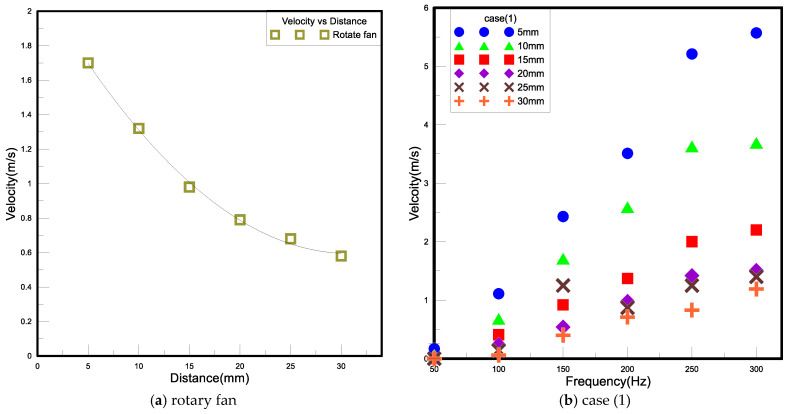

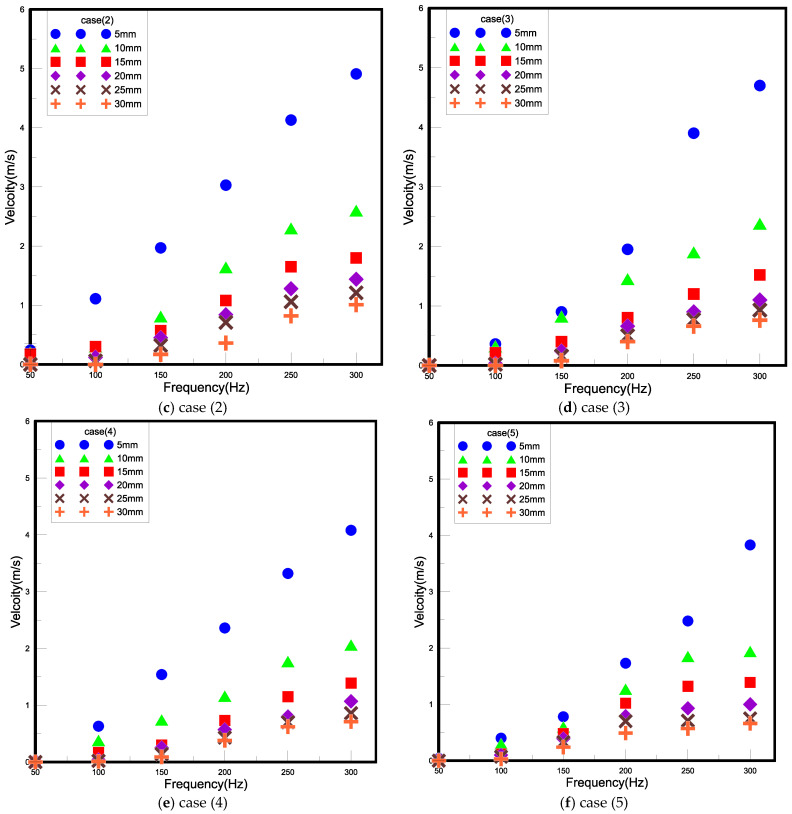
Each device when the wind speed and placement diagrams are under different frequencies.

**Figure 12:**
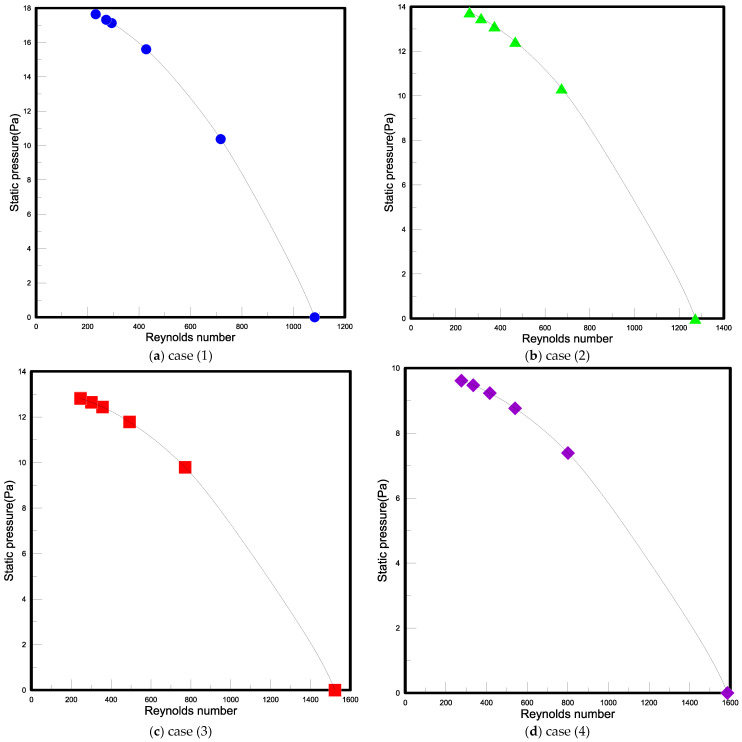

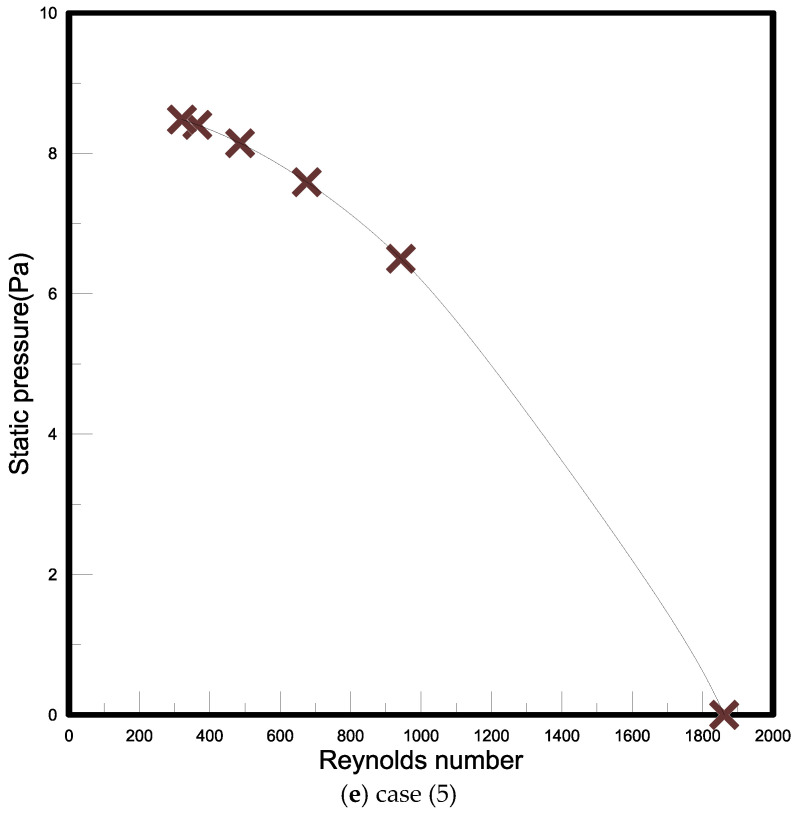
Each device of Reynolds number.

In general, the wind speed generated by the rotary fan and the closed PA is not high, and the distance between the placement position and the heat source is too close, which will make it difficult for the airflow to reach the heat source for heat exchange so that the effect of forced convection cannot be effectively exerted. This section is based on the strength of the wind speed as the basis for judging the heat dissipation effect, and the strength of the wind velocity disturbance can be regarded as an indicator of the turbulence strength. Figure 11a is the wind speed change measured by the rotary fan and the hot wire anemometer at different distances; Figure 11b–f is the closed piezoelectric jet fan with different external openings and the Wind speed measured by a hot wire anemometer at different distances. The measurement results show that the wind speed of the rotary fan and the piezoelectric fan is inversely proportional to the placement position. Among them, when the five cases are at 5 mm, the wind speed is the strongest. However, when the placement position is changed from 5 mm to 10 mm, the wind speed will increase rapidly due to the increase in the distance. However, when the placement position is between 10 mm and 30 mm, the distance of the wind speed decreases gradually, mainly because the eddy current is generated in this area, which drives the nearby air disturbance, so the measured wind speed will not vary greatly due to the increase in the distance; however, when the frequency is increased from 50 Hz to 250 Hz, the wind speed is proportional to the frequency, but when the frequency is increased from 250 Hz to 300 Hz, the wind speed is only slightly increased, indicating that the wind speed generated by the vibration frequency will reach the highest value. Figure 12a–e is represented by the Reynolds number; the Reynolds number of case (1)–case (5) is between 200 and 1800. Among them, in case (1), the static pressure is the highest, but the Reynolds number is the lowest; on the contrary, case (5) has the lowest static pressure and the highest Reynolds number, which shows that the main changing factor of the Reynolds number is the size of the outer opening. The higher the Reynolds number, the shorter the outer opening, and the lower the Reynolds number.

### 3.2. LED Thermal Performance Experiment

Taking a single LED as an example, its working temperature is about −40 °C to 80 °C, but the temperature of its light-emitting crystal is about 120 °C. High temperature has a great influence on the service life of the LED, so the temperature must be controlled below 80 °C. It takes about 20 min for the temperature of the LED module to reach a steady state, so the experiment takes every 25 min as the recording time. In this experiment, the supplied wattage is designed to be 1 W, 2 W, and 3 W. When the supply reaches 4 W, the temperature under natural convection exceeds 80 °C, so the supplied wattage will not be further increased. Since the power supply can only adjust the voltage and current, the wattage needs to be calculated and can only be measured with a relatively close wattage. The energy supplied by the LED is not completely converted into light energy. At present, the efficiency of the white LED light is about 30%, and the other 70% is in the form of heat. This part is divided into the finless type and finned type, of which the finless type is further divided into horizontal type, upward blowing type, and downward blowing type, to discuss the heat dissipation situation of the closed PA in different directions and using the traditional rotation fan dissipates heat under the same wattage. The thermal resistance and thermal conductivity are calculated according to the experimental results. The performance of the closed PA and the traditional rotary fan was evaluated by the thermal resistance and thermal conductivity. The closed PAs used in this experiment were operated under the same conditions, the ambient temperature was 23.5 °C, the input voltage was 30 V, the voltage frequency was 300 Hz, and the placement distances were 5 mm, 10 mm, 15 mm, 20 mm, and 25 mm. The change of each thermal resistance judges the effect of each heat dissipation method.

**Figure 13:**
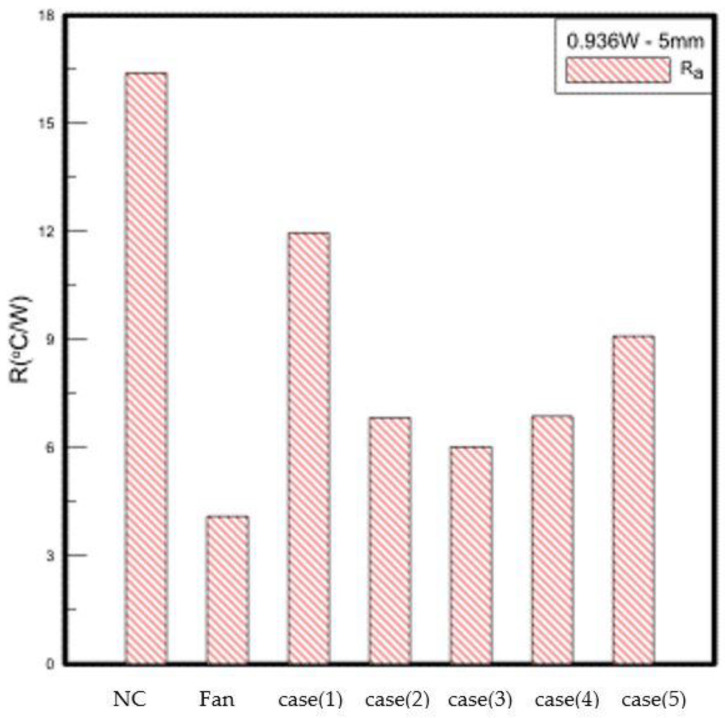
1 W LED experimental data.

In the experiment, it was found that when the closed PA is dissipating heat in a steady state, the central temperature is lower than the temperature on both sides. When the thermal resistance analysis is performed by using the diffusion thermal resistance, the calculation will generate negative thermal resistance, so in this part, the temperature of the three points is calculated. Take the average value and the convection thermal resistance calculated from the ambient temperature for analysis. The CH6 point measured under the rotating fan and natural convection is the ambient temperature, and the closed PA is measured at the exit temperature (One near the air outlet and one on the air outlet). 5 mm away from the heat source, the experimental results when the supply wattage is 1 W are shown in Table 3. Figure 13 is a comparison diagram of the thermal resistance of the 1 W placement position of 5 mm. The heat dissipation effect of the traditional rotary fan is better than that of the closed PA. It is excellent. Compared with natural convection, the temperature drops by about 31% to 32%. Although the heat dissipation effect of case (1) is the worst among the five forms, compared with natural convection, the temperature drops by about 9 to 11%; The temperature of case (2) and case (4) drops by about 20–24%; case(3) has the best heat dissipation of the five forms, and the temperature drops by about 22–26%, while case (5) is between case (1) and case (2), the temperature drops about 12–16%. In the experiment, it was found that the heat dissipation area of the five cases will be affected by the size of the opening. The temperature of the center point with a small opening is lower than that of the left and right sides. As the opening becomes larger, the temperature difference will decrease, or even the temperature of the center point will exceed the temperature on both sides. The air volume of (1) is concentrated at the CH2 point in the center, so it cannot have good heat dissipation for the overall LED; case (5) is because the outer opening is too large so that the air volume cannot be concentrated, then case (3) is the best opening size. The closed PA has zero mass flux, and it will be sucked in and discharged at the same time. If it is too close to the heat source, during the vibration process, the fluid around the heat source or the fluid that has been discharged to the outer flow field will inevitably be sucked back into the cavity, which will not only affect the fluid flow during discharge but also bring the fluid with a higher temperature near the heat source back into the cavity. As time increases, the temperature of the fluid in the cavity will increase, which will not be conducive to heat dissipation. When the distance from the heat source is 10 mm, the temperature of the rotary fan after heat dissipation is not much different from the temperature of 5 mm. The closed PA has a slight improvement in the heat dissipation effect, mainly because the measurement distance increases, and the PA is in the vibration process. It can suck in the cold air on both sides of the outlet and push the air to the heat source for heat exchange.

**Figure 14:**
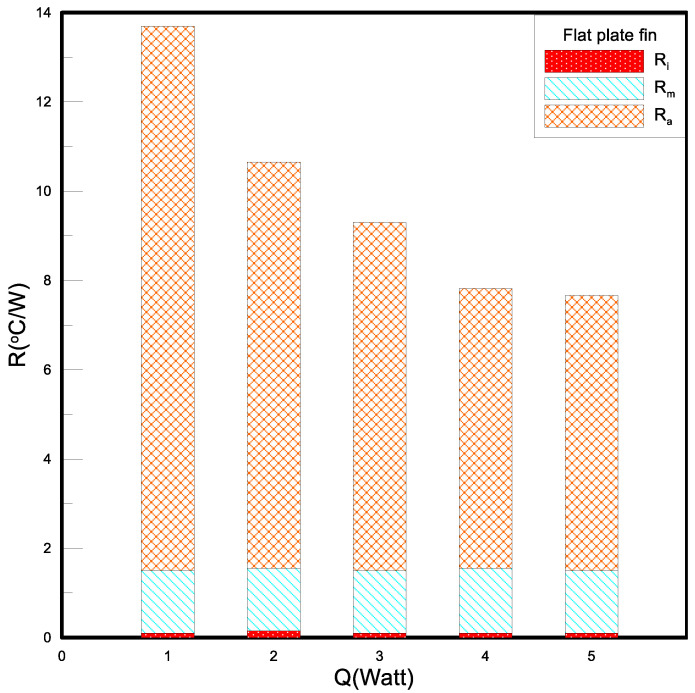
Thermal resistances of nature convection.

After the LED heat dissipation die is assembled with fins, the supply wattage is increased to 1–5 W. The experimental results under natural convection are shown in Figure 14. The thermal contact resistance (Ri) is the factor between the LED substrate and the heat dissipation fins. The gap makes it difficult for thermal convection, thus, forming thermal resistance. This thermal resistance is not directly related to the wattage supplied and the heat dissipation method. Generally, it can be applied by pressurizing or applying a thermally conductive medium to the gap. The material used in this experiment is a thermal paste, and to reduce to avoid experimental error, try to control the contact thermal resistance within a fixed value, and the contact thermal resistance measured in the experiment is between 0.08–0.15 °C/W. The material thermal resistance (R_M_) is the thermal resistance caused by the material and the thickness of the material itself. The experimental results show that the material thermal resistance of the flat fin is about 1.4 °C/W. After adding heat dissipation fins, the LED heat dissipation module does have a cooling effect. The temperature of the LED heat dissipation module with flat heat dissipation fins at 5 W is 61.8 °C, which is higher than the temperature when no fins are installed to provide 3 W. Low, it mainly strengthens the convection effect and reduces the convection thermal resistance (Ra).

## 4. Conclusions

This paper is devoted to designing a new type of closed PA as a cooling fan and discussing the input conditions. According to the different parameters, such as piezoelectric sheet spacing and opening area, five groups of closed PAs are made. The research results show that the better type design and operating conditions can enable the fan to give full play to its performance, and the heat dissipation effect is better than that of the traditional rotary fan, which is a new way of heat dissipation. The summary is divided into two parts for discussion. The first part is the results of type design and performance analysis, and the second part is the experimental results of LED module heat dissipation. (1) It can be known from the experiment that the wind speed, displacement of the piezoelectric sheet, and noise of the closed piezoelectric jet fan are proportional to the frequency, but considering the noise factor, the frequency is set to 300 Hz for heat dissipation experiments. (2) The displacement of the piezoelectric sheet will change with the supply voltage frequency, which is roughly proportional. When the supply frequency is 100 Hz, 250 Hz, and 400 Hz, the displacement of the piezoelectric sheet will decrease, indicating that the supply frequency Resonance cannot be achieved with the natural frequency of the piezoelectric sheet. (3) The size of the opening will affect the flow rate. A large opening can provide stronger air volume, but the wind speed is low; a small opening can increase the jet airspeed, and the air volume is weak. Case (3) is a form of both air volume and wind speed. (4) The volume of the enclosed piezoelectric jet fan is 8100 mm^2^, which is conducive to placing it in electronic products for heat dissipation. (1) When the closed piezoelectric jet fan is placed too close to the heat source, the high-temperature fluid around the heat source will be sucked back, causing the temperature of the fluid in the cavity to rise and reducing the heat dissipation effect. Therefore, the fan should be kept 10–20 degrees from the heat source. The distance of mm reduces the temperature of the fluid sucked into the cavity and achieves a better heat dissipation effect. If the distance is too far, the enclosed piezoelectric jet fan cannot send the air to the heat source, nor can it effectively dissipate heat. (2) According to the experiment, in the heat dissipation experiment without fins, it is found that case (3) has a good heat dissipation capacity. When the placement position is 15 mm, the heat dissipation effect capacity is 120% of that of the traditional rotary fan. (3) After adding heat dissipation fins, the LED can effectively reduce the temperature, which can increase the wattage by 3–4 watts. Coupled with the closed piezoelectric jet fan for heat dissipation, it can be increased by about 2–3 watts. The piezoelectric jet fan will still suck the high-temperature fluid between the fins due to the short distance, which is the same as the situation without fins. (4) When the heat dissipation device is equipped with a single cooling fin or is replaced with a combined piezoelectric jet fan, its optimal placement will be affected. The best placement of the closed piezoelectric jet fan is 15–20 mm, and in the case of adding fins at the same time, the placement position is 30 mm or later. (5) Find out the empirical formula of case (2)–case (5) by dimensional analysis, and its error value is within 28%, which shows the reliability of this empirical formula.

From the comparison results of power consumption, it can be seen that the power consumption of the closed PA in the same reduction of 1 °C is only 10–20% of that of the rotary fan, and the cost is low while achieving low cost and low energy consumption. The principle of the closed PA studied in this paper is similar to that of the cantilever beam piezoelectric fan, but through the difference in structure and material, a new heat dissipation mechanism has been invented, and its heat dissipation efficiency is better than that of the traditional rotary fan in some cases. In terms of power consumption, the closed piezoelectric jet fan is indeed lower than the traditional rotary fan, which can effectively achieve energy saving. In terms of volume, the closed PA is thin and low in cost, and a new invention patent has been applied for.

## Figures and Tables

**Table 1:** Specifications of closed PA devices.

Case No.	Diameter (mm)	Cavity Volume (mm^3^)	Spacing d_c_ (mm)	Inside Length L_inside_ (mm)	Outside Length L_outside_ (mm)	Opening Area (mm^2^)
Case (1)	41	2268.2	2	4	6	12.5
Case (2)	41	2268.2	2	4	8	15
Case (3)	41	2268.2	2	4	10	17.5
Case (4)	41	2268.2	2	4	12	20
Case (5)	41	2268.2	2	4	15	23.75

**Table 2:** The revised noise decibel.

Object	Before (dB)	After (dB)	Revised Percent (%)
Rotating Fan	51.2	50.6	1.17
case (1)	52.8	52.4	0.76
case (2)	51.3	50.8	0.97
case (3)	50.1	49.4	1.39
case (4)	50.8	50.2	1.18
case (5)	52.1	51.6	0.96

**Table 3:** Temperatures of LED for free and forced convection under 1 W and 5 mm.

Flow Patterns	Temperatures of LED			
	CH1	CH2	CH3	CH6
Natural Convection	39.6 °C	40.5 °C	39.6 °C	23.5 °C
Rotate fan	27.5 °C	27.8 °C	27.5 °C	23.5 °C
case (1)	36.5 °C	35.9 °C	36.6 °C	24.4 °C
case (2)	31.3 °C	31 °C	31.2 °C	24.3 °C
case (3)	30.6 °C	29.8 °C	30.6 °C	24.3 °C
case (4)	31.0 °C	30.5 °C	30.9 °C	23.9 °C
case (5)	34 °C	34 °C	34 °C	23.8 °C

## Data Availability

All data are offered by the authors for reasonable request, and the novel device of the piezo actuation jet is available from the authors.
